# A qualitative study about the gendered experiences of motherhood and perinatal mortality in mountain villages of Nepal: implications for improving perinatal survival

**DOI:** 10.1186/s12884-018-1776-3

**Published:** 2018-05-15

**Authors:** Mohan Paudel, Sara Javanparast, Gouranga Dasvarma, Lareen Newman

**Affiliations:** 1 Initiative for Research, Education and Community Health-Nepal, Kathmandu, Nepal; 2 Southgate Institute of Health, Society and Equity, Flinders University, Adelaide, Australia; 3 College of Humanities, Arts and Social Sciences, Flinders University, Adelaide, Australia; 4 Education Arts and Social Sciences Divisional Office, University of South Australia, Adelaide, Australia

**Keywords:** Motherhood, Perinatal survival, Mountain, Childbirth, Gender, Nepal

## Abstract

**Background:**

We aim to examine the gendered contexts of poor perinatal survival in the remote mountain villages of Nepal. The study setting comprised two remote mountain villages from a mid-western mountain district of Nepal that ranks lowest on the Human Development Index (0.304), and is reported as having the lowest child survival rates in the country.

**Methods:**

The findings are taken from a larger study of perinatal survival in remote mountain villages of Nepal, conducted through a qualitative methodological approach within a framework of social constructionist and critical theoretical perspectives. Data were collected through in-depth interviews with 42 women and their families, plus a range of healthcare providers (nurses/auxiliary nurses, female health volunteers, support staff, Auxiliary Health Worker and a traditional healer) and other stakeholders from February to June, 2015. Data were analysed with a comprehensive coding process utilising the thematic analysis technique.

**Results:**

The social construction of gender is one of the key factors influencing poor perinatal survival in the villages in this study. The key emerging themes from the qualitative data are: (1) Gendered social construct and vulnerability for poor perinatal survival: child marriages, son preference and repeated child bearing; (2) Pregnancy and childbirth in intra-familial dynamics of relationships and power; and (3) Perception of birth as a polluted event: birth in *Gotha* (cowshed) and giving birth alone.

**Conclusions:**

Motherhood among women of a low social position is central to women and their babies experiencing vulnerabilities related to perinatal survival in the mountain villages. Gendered constructions along the continuum from pre-pregnancy to postnatal (girl settlement, a daughter-in-law, ritual pollution about mother and child) create challenges to ensuring perinatal survival in these villages. It is imperative that policies and programmes consider such a context to develop effective working strategies for sustained reduction of future perinatal deaths.

**Electronic supplementary material:**

The online version of this article (10.1186/s12884-018-1776-3) contains supplementary material, which is available to authorized users.

## Background

The perinatal period is considered critical in terms of saving the lives of mothers, preventing stillbirths and neonatal deaths as well as in ensuring a healthy future generation [1]. Perinatal deaths include pregnancy losses after 22 weeks’ gestation (stillbirths) and neonatal deaths within the first seven days (week) after birth [2]. Measurement of perinatal mortality within this window is commonly used for international comparisons of mortality rates. An extended perinatal period that includes stillbirths plus neonatal deaths within the first 28 days (month) after birth, is also in use, such as that reported in US and Australian studies [3, 4]. The extended perinatal period is useful to monitor and examine deaths across the gestation age spectrum, and this is applied in the present paper unless otherwise specified separately as stillbirth or neonatal death.

Prevention of perinatal deaths is a global public health priority. In 2015, the global stillbirth rate was 19 per 1000 births [5] and the neonatal mortality rate was 21 per 1000 livebirths [6]. Worldwide, more than 14,500 perinatal deaths are reported daily, comprising both stillbirths [7] and neonatal deaths [8]. In 2015 neonatal deaths comprised almost half (45%) of global under-five deaths and 75% of all infant deaths [8]. Of the total neonatal deaths, more than two-thirds occur within the first week of life (early neonatal deaths). It is also known that almost all perinatal deaths (98%) occur in developing countries; 75% in Sub Saharan Africa and South Asia [1, 9]. With current rates of reduction, it is estimated to take more than 100 years for women living in South Asia and Sub Saharan Africa to achieve a perinatal survival rate similar to that of women in developed countries [1].

Poor perinatal survival is one of the major public health problems in Nepal. Although Nepal is one of the few developing countries to have achieved the United Nations Millennium Development Goal of reducing under five mortality by two-thirds between 1990 and 2015 [10], its perinatal mortality rate is still very high compared to infant and under-five mortality rates. Nepal records more than 30,000 perinatal deaths annually [10]. The most recent data on child mortality at the time this study commenced are from the 2011 Nepal Demographic and Health Survey (NDHS 2012), which show the perinatal mortality rate for Nepal was 37 per 1000 total births (stillbirths and early neonatal deaths within 7 days after birth combined) while the neonatal mortality rate was 33 per 1000 livebirths [11]. These rates are unequally distributed with Nepal’s mountainous region having one of the highest such rates in the world - a neonatal mortality rate of 46/1000 livebirths and a perinatal mortality rate of more than 40 per 1000 total births (stillbirths and early neonatal deaths ie within the first week after birth), which are equivalent to the highest rates in Sub Saharan Africa [6]. Nepal’s demographic survey report does not currently include an extended perinatal mortality rate (stillbirths plus neonatal deaths within the first month after birth), and with these missing deaths incorporated Nepal would likely have a significantly high extended perinatal mortality rate.

Globally, there is abundant literature on the epidemiology of perinatal deaths: the “when, where and why”, with a list of proven interventions to improve perinatal survival [1, 9, 12–16]. However, these studies explain perinatal survival predominantly from a bio-medical standpoint, identifying prematurity, asphyxia and infection as the main causes of death [1, 9]. The influence of gender and social determinants, which are widely discussed in relation to maternal [17] and under-five child mortality [18], have been scarcely discussed in relation to poor perinatal survival.

Gender is one of the major social determinants of health [19], influencing reproductive behaviour, healthcare access and perinatal survival. Gender includes socially and culturally constructed roles that men and women play, and the relations that arise from such roles [20]. An examination of gendered contexts comprises studies of different behaviour, roles, responsibilities and expectations of men and women and their influence on health and sickness responses. Gendered roles and responsibilities shape access/resources, which in turn are shaped by gender based norms and values. Gendered perspectives have been discussed mostly in relation to the survival of older children and pregnant women [17, 18, 21]. For example, male children have a 20% higher chance of survival during the first and the fourth years of life in Nepal, India, Bangladesh, Pakistan and Vietnam [22]. A range of studies from South Asia discusses gender as an important factor impacting maternal and child survival [21–25]. However, few studies describe gendered constructions and their influences, particularly on perinatal survival.

Perinatal survival is often considered technically as requiring improvements predominantly in the medical aspects of perinatal deaths. The *Lancet* papers attempted to demystify the myth that only highly technical medical solutions can reduce perinatal deaths [26–28]; however, the suggested interventions still remain medically based. The impact of the gendered cultural context of pregnancy and childbirth, which this paper aims to examine, has not been well considered in remote Nepal other than the mere statement of an aim for gender equality in Nepal’s policy related documents [29, 30]. The literature about perinatal survival discusses mainly sex-specific differentials of stillbirths and neonatal mortality, often concluding higher survival chances of female babies [31, 32]. While discussing perinatal survival, the above mentioned studies lack consideration of how gendered aspects before and during pregnancy and childbirth impact perinatal survival outcomes in pregnancy and after birth. Some studies report female foeticide in India [33] and China [34], the impact of differential care seeking for newborn girls in Bangladesh [35], and greater risk for baby girls during the late neonatal period in the southern plains region of Nepal [36]. Still, an in-depth examination of the gender and cultural contexts of perinatal deaths has not been a focus in any of these studies.

After the International Conference on Population and Development (ICPD), held in Cairo in 1994, the gendered position of woman was acknowledged as crucial in personal health and overall reproductive health including in decisions regarding when to marry, and whether, when and how many babies to have [37]. However, women and children have continued to suffer from biased gender norms and relations in many ways. The child’s sex is found to intersect with fertility behaviour; there are closely spaced pregnancies in places where there is a strong preference for male children. It is estimated that family planning (spacing, limiting births and delaying) alone could avert 60% of maternal and 57% of child deaths worldwide [38]. Unintended pregnancies and pregnancies at young age produce significant risks of maternal as well as neonatal and child death [39]. In many developing countries, women’s access to care during pregnancy, childbirth and the postnatal period is influenced by the decisions of their mother-in-law and husband [17, 40]. A previous study in Nepal’s capital Kathmandu found that men’s and women’s cultural positions impeded the uptake of biomedical birthing care, while two other studies found that mothers-in-law play significant roles: in Kathmandu Valley they negatively influence access to pregnancy care, but in the remote areas positively influence birth preparedness and safety [23, 24, 41].

Although information about the patterns and distribution of perinatal mortality is available from various studies in both the hills and plains regions of Nepal [42–53], studies from the mountainous regions are rare. The available studies either describe medical causes of perinatal deaths [49–53] or merely report the pattern of health service utilisation and mortality outcomes [42–48]. These studies are mostly cross-sectional surveys or further analysis of reports of national demographic and health surveys. In general, in-depth, qualitative studies examining poor perinatal survival are lacking in Nepal. Moreover, although it is known that the mountainous region has the highest death rates and the lowest utilisation of pregnancy, childbirth and postnatal services by women, a socio-cultural explanation is lacking behind these high perinatal mortality rates and low service uptake in the mountainous region. In this regard, the present in-depth qualitative study adds to the literature by investigating the gendered contexts of poor perinatal survival in Nepal’s mountain villages.

## Methods

### Study design and setting

We conducted a qualitative study utilising social constructionist and critical theoretical approaches to examine the gendered cultural contexts of poor perinatal survival in the mountain villages. Social constructionism and critical theoretical perspectives fit with the study’s socio-cultural approach and guided the choice of research method—policy review and qualitative interviews, analytical technique—the inductive thematic analysis, and discussion that revolves around gender, which is seen as a structural force underlying the ongoing poor perinatal survival rates in the mountain villages. A qualitative approach allowed the first author to listen to the voices of unheard, silenced and marginalised groups [54, 55], ie of the vulnerable ethnic women losing their babies. This also allowed an understanding of the participants’ experience in a much more considerate way than the structured positivist approach of quantitative surveys [55]. The study’s methodological approach also fits the objective of examining the gendered cultural construction of poor perinatal survival by soliciting the experiences of women and their families about why they think they experienced perinatal deaths, their beliefs and their care seeking behaviour for mother and baby. The study was conducted in Mugu district, one of the remote mountain districts with the lowest Human Development Index (0.304) [56], and reportedly the lowest child survival rates in the country [11].

### Data collection and participant recruitment

The interviews were conducted by the first author**—**a Nepali national with seven years’ experience of working in the health field in Nepal. The participants comprised 42 women (aged 16–35) and their families who had experienced at least one neonatal death or a stillbirth in the last four years **(see** Additional File 1**).** The women’s interviews were supplemented by interviews with 10 nurses/auxiliary nurses, two female health volunteers, two support staff, one Auxiliary Health Worker, one traditional healer and four stakeholders (two local journalists and two activists from local non-governmental organizations working in child rights and the prevention of child marriages). These primary data were supplemented by a review of six key Nepalese policy documents related to perinatal survival and field notes taken during the fieldwork (February to June 2015). The in-depth interviews **(see** Additional file 2**)** were considered crucial to explore in detail the phenomenon of perinatal mortality [57, 58].

Participant recruitment started by purposively selecting the district, and selection of two study villages after consultations with the District Health Office. The religio-cultural composition, relatively larger population size and availability of childbirth service in the chosen villages provided a community lab to study the socio-cultural contexts of poor perinatal survival. The first village had approximately 4000 people following Hindu religious beliefs, and the second village about 3000 people of Hindu faith as well as those *(Lama)* following Buddhist beliefs. Both villages had access to local health facilities, and birthing units within one hour walk. The first village had also an access to district level hospital within one hour walk, while the hospital was at about seven hours’ walking distance from the second village. The villagers live in densely populated small settlement areas, in mud and stone huts shared together with cattle on the ground floor, locally described as *Gotha*. These huts/households are often adjoining with each other.

Participants were recruited via female health volunteers, a social mobiliser and a health worker as gate keepers. Women and families were informed via these gate keepers to contact them if they had recently experienced a perinatal death (in the previous four years), and wished to be part of this study. The gate keepers were locally trusted individuals who had opportunities to inform women and families during their everyday contacts in the neighbourhood, such as while fetching water or walking to and from the forest. The gate keepers provided a list of women willing to participate in the study, which was reviewed by the first author, who made further contact with eligible participants to confirm their participation. Recruitment continued until saturation, ie participants ideas started repeating and no specific new idea emerged, which was decided based on the first author’s repeated listening to interview records and field notes. Local health workers, nurses/auxiliary nurses, female health volunteers and local stakeholders were interviewed to supplement women’s interviews to include their rich day to day experiences related to childbirth and perinatal survival. As Devers and Frankel [59] have described, utilising the gate keepers as recruiters created trust in the villagers. Some participants were also recruited via other participants’ contacts (snowballing). All interviews were digitally recorded with the permission of the participants, and before the start of each interview, written informed consent was sought from each participant.

### Data analysis

The interview records were simultaneously translated into and transcribed in English by the first author. Six random transcripts were checked by five bilinguals (Nepali and English) experts who were current and past PhD students of Flinders University. During the fieldwork, the first author immersed himself in the recordings, noted key local terms and any queries, and verified these with participants. The data were analysed using thematic analysis techniques proposed by Braun and Clarke [60], and organised using the qualitative data analysis software NVivo Version 10:00, that also facilitated the coding process. The themes are the key concepts emerging from the data, which in this study relate to gender and cultural contexts and perinatal survival in the mountain villages. The analysis followed Braun and Clarke’s six phases: familiarisation with the data including transcribing; coding; searching for themes; reviewing themes; defining and naming themes; and reporting findings. The process from coding to theme generation was closely scrutinized by the three supervisors and any inconsistencies in code generation and theme development were settled by discussion in monthly meetings during the larger Ph.D. project [61] on which this paper is based.

### Ethical considerations

This study went through ethics approval as per Australia’s National Health and Medical Research Council (NHMRC), and followed the NHMRC’s universal standards of research ethics [62]. Approval was sought from: (i) the Social and Behavioural Research Ethics Committee of Flinders University; (ii) the Nepal Health Research Council (NHRC); and (iii) the District Health Office of the study district. Voluntary and informed written consent was sought from all participants.

## Results

Three key themes emerged regarding the influence of the gender-related cultural contexts of motherhood experiences that lead to poor perinatal survival in these villages. These themes are: (1) Gendered social construct and vulnerability for poor perinatal survival: child marriages, repeated child bearing and son preference; (2) Pregnancy and childbirth in intra-familial dynamics of relationships and power; and (3) Perception of birth as a polluted event: birth in *Gotha* (cowshed*),* and giving birth alone. Each theme is now discussed in turn.

### 1. Gendered social construct and vulnerability for poor perinatal survival: Child marriages, repeated child bearing and son preference

This study found that in the villages studied in this research, the construction of *‘Gharbar (girl settlement)’* has been a major driver of early marriages of girls, early and repeated childbearing and baby losses. *Gharbar* is a gendered construction by which it is perceived that a girl is secured and settled when she is married, gives birth early and has a surviving child, preferably a baby boy.

#### 1.1 Child brides, child mothers and perinatal deaths: A common phenomenon

Nepal’s minimum legal marriage age is 20 years for both females and males. However, it is common practice, especially in remote areas, to continue the tradition of getting daughters married well below this age. As such, they become child brides and child mothers. In these villages early marriage, early childbirth and subsequent perinatal deaths were common among the majority of participants. A majority of the women (38 out of 42) were married and had their first birth before age 18, with 10 of the 42 having been married at or before age 14. Early marriage was not considered a matter for concern, with most participants not expressing any negative feelings about this. A 16 year-old mother who gave birth last year and lost her first newborn considered it normal and common in the village:

*I gave birth at 16 [married at 14]. I don’t think I am very young to give birth. Others gave birth at 14 and 15 too. (16 year-old mother)*

Only rarely did a participant regret early marriage when they were unable to continue their education and they also lost a baby. Another young mother, who was married at 16, and lost a newborn this year stated:

*I pray to my Dewata (God) not to bring to anyone the situation like mine. Let everyone save his or her babies. I got married too soon (Bhagi Bibaha), disturbed my education, and at the end lost my baby. I am feeling alone. (18 year-old mother)*

This young mother went through *Bhagi Bibaha*, a more recently introduced system in the local culture of marriage at a later age than that done traditionally – i.e. among young adolescents rather than the traditional engagement in childhood, but still well below the minimum legal marriage age for girls. Among the participants there were many others who had similar experiences of *Bhagi Bibaha*, early births and subsequent loss of a baby.

#### 1.1.1 Transition in marriage practices, but child marriages still continued

Marriage practices have changed in many parts of Nepal, with rising age at marriage for both males and females, particularly among urban residents and educated females, for whom the average marriage age has now reached age 20 or older [63].There is also an increasing trend of love marriage (marriages arranged by the bride and groom themselves, similar to in western societies) in the plains as well as the hills areas, but child marriages have not stopped.

Until about 25 years ago, there was consensual marriage with a parental cultural contract, similar to an engagement, between the bride’s and groom’s families – locally called *Tike Bibaha* – when the children were very young*,* and usually when a daughter reached age 7. A formal cultural ceremony then occurred at about 11 years old, when the girl was perceived to be ready to leave her parents’ home. Over the last few years such practice has changed from early age cultural contracts to *Bhagi Bibaha*, a growing culture of marriage at early and mid-teenage years, held usually without a cultural ceremony and often kept secret from the police and local political activists, as these marriages contravene the legal minimum marriage age of 20 years. The boy and girl meet each other briefly, and the girl goes to stay at the boy’s house. They are then accepted as a formally married couple. One of the local community health workers said:

*The national government introduced the Child Marriage Act. According to this, no one should marry before [age] 20. However, this doesn't work here. Before, there used to be marriages after early age parental contracts (Tike Bibaha), now it turns into secret marriages of young adolescents without a cultural ceremony (Bhagi Bibaha). (Local community health worker, HSP12)*

*Bhagi Bibaha* is simply a way of getting young adolescent girls (and boys) cohabiting and having children early without the process of *Tike Bibaha*, which may be banned under the new marriage act. But the boys and girls start cohabiting at the same age as they had gone through the traditional process of *Tike Bibaha* and the subsequent ceremony of the girl leaving her parental home at or just before puberty. Interviews revealed that some parents even move to nearby forests and farmland at high altitudes *(Lekha)* to secretly arrange the marriage of young daughters.

The perception of an adolescent girl as ready for marriage and childbearing after her first menstruation and factors such as encouraging *Bhagi Bibaha* with less parental control on the adolescents, are more common in lower castes:

*Adolescents from the lower caste community meet for local folk singing and dancing (Deuda) from young ages. They are perceived ready to marry and give birth after their first menstruation, and this also allows more freedom to participate in singing and dancing. I think that is a reason for child marriage. They know each other and end up with Bhagi Bibaha. (Senior Auxiliary Nurse, HCM1)*

*Bhagi Bibaha* is common also because it is financially less burdensome on both the boy’s and the girl’s families than traditional arrangements. For the girl’s family, the dowry practice is commonly seen as a serious issue for a daughter’s marriage in the southern plains region of Nepal [64], and is less common practice in *Bhagi Bibaha*. Another finding is that to avoid legal punishment, these adolescent marriages are usually not registered officially. Similarly, a child’s birth and death occurring as a consequence of such early marriage are also not registered:

*They have already had children. They do not register births and deaths of their babies. Neither are their marriages registered. They go for registration only when they are above [age] 20. (Local journalist, SH9)*

#### 1.1.2 ‘Gharbar (girl settlement)’: A driving motive behind child marriage, early childbearing and repeated pregnancy

It has been mentioned earlier that a girl is perceived secured and settled when she is married, gives birth early and has given birth to a baby, preferably a boy. Participants described settling their daughters down with a home and husband– known locally as *Gharbar* (girl settlement) – as a driving force for early marriage. To settle a home, young girls get pregnant and bear children frequently, particularly those who experience baby losses. One mother described how she became pregnant with birth intervals (every year) when she lost three pregnancies (2 neonatal deaths and 1 stillbirth). Within five years of marriage, she is now pregnant with her fourth child.

*First time my baby was born dead. Last year, I gave birth to another baby boy, but the baby died. Therefore, I became pregnant soon after this. This year I gave birth a third time, another baby boy; this died too. If both of these babies had survived, one would have been able to walk around, and another could have been sitting and standing on support. Therefore, I became pregnant soon after (Khata Khattai Sutka Base). (20 year-old mother)*

Parents have a drive to ensure their daughters’ *Gharbar* because it is essential for marriage survival or preventing detachment/divorce from their husbands. The parents are influenced by everyone’s concerns including relatives, neighbours and peers. One local stakeholder described the parents’ concern as follows:

*…unless there is a child [born], it is not considered a Gharbar (girl settlement). Parents become anxious about whether or not their daughters will give birth sooner. This culture puts pressure on young couples to give birth sooner. (Local Stakeholder, SH8)*

- ***➢ Gharbar in the context of high perinatal mortality***

The uncertainties surrounding babies’ survival is related to the young women’s *Gharbar*. Parents’ feeling of uncertainty whether their daughter will produce a child is clearly evident in a young father’s interview, who postponed the rituals such as *Chhaith,* normally celebrated on the sixth day after the birth of a child:

*Last year, it was just a big expense during the Chhaith. The baby did not survive. We are not sure whether this baby will survive. We have also postponed Chhaith this time. (21 year-old father)*

Although the women and their families have a level of acceptance of perinatal death as their *Karma* (results of past deeds) and fate, after the early loss of a baby the bereaved mother (the woman losing the child) comes under pressure from her parents and in-laws to conceive another baby quickly. Thus, for the sake of *Gharbar,* the young women are caught in a vicious cycle of child marriage leading to early and repeated childbearing. Some women, after repeatedly losing babies, felt neglected by their husbands and in-laws and returned to live in their maternal family. Thus, giving birth, preferably to a boy which survives, is essential to save a woman’s marriage. The participants in this study postponed using any family planning method until babies were about four years old. Many did not even buy clothes for newborns as they were not sure whether the babies would survive.

- ***➢ Gharbar related to gendered religio-cultural norms***

The drive towards a girl’s *Gharbar* is a result of gendered religio-cultural norms. It is believed that ensuring a daughter’s *Gharbar* is a good *Karma* that earns God’s blessings *(Punya).* It is believed that if daughters are married *(Kanyadan)* before menstruation, it will bring more *Punya* to the parents. The parents who can see their great-grandchildren *(Panati)* are believed to be blessed with more *Punya* during their lifetime and reach heaven after death. One of the local journalists noted:

*Child marriage is very common in this village. It is everywhere in this district. Here, people say if parents marry their daughters earlier (Kanyadan), before their menstruation, they will earn good Karma (Punya Dharma) and will be more blessed from Dewata (God). (Local Journalist, SH9)*

There is a similar pressure for early childbearing from in-laws:

*I have no grandchildren yet [she looks sad and uncertain]. Dilma gave birth to a baby two years ago, but the baby didn’t survive. And, last year another birth, but the baby didn’t survive again. My younger son got married this year, but his wife is not yet pregnant. (Dilma’s mother-in-law, 61 year-old woman)*

Marriage and birth of a baby are also considered as a test of femininity *(Naritwa)* as well as masculinity *(Purushatwa).*

*They want to have a baby as early as possible. Women feel that now they are mothers (Aama/Matritwa). Men feel that now they have proven their masculinity (Purushatwa). (Local teacher, SH4)*

#### 1.2 Son preference, repeated pregnancy and perinatal deaths

Son preference is a deeply rooted gendered construction in the villages, contributing to repeated pregnancies and increased perinatal mortality. This has widespread ramifications ranging from sex detection and pregnancy termination of a girl foetus, to neglecting a baby girl and a woman. A local female activist narrated the story of a village woman who had 12 pregnancies in 17 years of marriage in the hope of bearing a baby boy. The woman lost four baby girls:

*She gave birth to 12 girls. This time, I went to see her and found that she is pregnant with the 12* ^*th*^ *child. Of the total, eight girls survived and four died. I told her, ‘why do you continue giving births these many times?’ She replied, ‘Madam, we are afraid of breaking our family succession (Aputo). We went to a faith healer, worshipped Dewata (God), and changed our house. What to do, I didn't have a boy yet’. (Woman activist from local Women Development Office)*

The strong preference for boys has influenced many women to have repeated pregnancies with close spacing. Twenty-five year-old Hashakali has two surviving daughters and one surviving boy, two stillbirths and three abortions (two sex-selective and one spontaneous abortion). It was evident that Hashakali experienced high-risk pregnancies, even with a prolapsed uterus, in order to have more boys:

*We thought to have one more boy, and do the operation (Minilap*—*the female sterilisation method). But, now it seems that we couldn't have another boy yet. I have only daughters.*

*… You know, I don't want to hide this. I have prolapsed uterus (Aangjhadne). If I carry any load of firewood or grass or any heavy thing, I feel it coming out, bulging out. It is burning and painful. I had this during this young daughter's birth. I suffered a lot while giving birth to this daughter. I suffered again badly during the last birth (the stillbirth). (25 year-old mother)*

The study participants also postponed using contraceptives until they had baby boys. A 37-year old father with three daughters, who already lost two baby boys, stated:

*We have only girls. I was expecting a boy from her, but it is not yet possible. My wife is also not using any contraceptive [Suhi: injection medroxyprogesterone] for this purpose. (37 year-old father)*

Conceiving repeatedly soon after giving birth was their strategy to have a surviving boy child as quickly as possible:

*The elder sister-in-law is using contraceptives [Suhi: injection medroxyprogesterone]. The younger sister-in-law and I still have young boys. We will do it once they grow up [to about 4 years old]. (25 year-old mother)*

This context of strong son preference has created a sense of fear among women if they have not yet had a boy because the mothers as well as newborn girls are likely to be neglected and ignored after birth. Nurses related that women and their visitors were reluctant to embrace and breastfeed newborn girls. Couples without a son feel stigmatised in the villages and are called *Aputo/e* (almost infertile)*,* which makes them feel sad. A woman who already had a stillbirth and a neonatal death described this:

*You know, villagers make you feel down if you have only girls. My husband is bullied by his friends and neighbours for not having a boy yet. We need a boy. I have seen how badly others treat you when you have only daughters. This is embarrassing. I feel worried about it. In my last pregnancy, we went to Nepalgunj [a nearby city] to identify our baby’s sex. It was a girl; we had to abort it. (25 year-old mother)*

### 2. Pregnancy and childbirth in intra-familial dynamics of relationships and power

The villagers did not consider pregnancy and childbirth as special events needing additional care and support. Such consideration is related to intra-familial dynamics of relationships and power with respect to the unequal social position of being a woman in general, and a daughter-in-law in particular. A daughter-in-law has the lowest position in the family hierarchy and decision making (Luitel, 2001).

#### 2.1 Work is the priority: Pregnancy and childbirth are not special events

Many families did not perceive that pregnancy needs additional care, rest, food or preparation for birth. During the fieldwork, women, especially daughters-in-law, were observed as always being on the move for day-to-day work collecting firewood, fodder and grass from the forest, and grazing cattle. It takes as long as three to six hours to return home after collecting firewood, fodder or grass from the forest, which are routine jobs for women without exemption, even during the last weeks of pregnancy. Indeed, there were many reports of women giving birth in the farmland, or on their way to/from home in the forest:

*I gave birth at millet farm (Kodebari). I looked around if anyone was there. I saw my cousin at a distance, ploughing his farmland. I got a sickle (Baso) from him to cut the baby’s cord, and I then made a thread from a piece of my shawl (Sal) to tie my baby’s cord (Navi). When I had birthing pain, I thought to go back home and took a few steps, but the pain increased and I couldn't walk further. I gave birth on the farm. Later [after about an hour], I wrapped the baby with my Sari and returned home. (27 year-old mother)*

One participant narrated a story about a neighbour who gave birth in the field and became unconscious after giving birth:

*She had gone to farmhouse (Lekha) to harvest potatoes. She was alone there. After birth, she became unconscious. The baby was lying on the field, nobody to cut the baby's cord (Navi). The baby was already dead. (16 year-old mother)*

Some participants cited work exhaustion *(Tapa)* and falls causing baby losses, particularly stillbirths. A mother who already experienced several stillbirths, neonatal and toddler deaths, described her last pregnancy:

*I had three falls during my last pregnancy. I think due to this Tapa (exhaustion) I lost my baby in the last month of pregnancy (Hunemahina). (32 year-old mother)*

Another mother had a similar story of falling during pregnancy which ended with a stillborn baby in the eighth month:

*I was chasing my cow, trying to return it to farmhouse (Lekha). On the way, the cow ran away and entered into our local river…Water current in the river was strong. … I [lost my balance and] fell into the river. I was badly hurt. I became sick for one month. Then I gave birth, the baby was dead, looked thin and dry, and baby’s cheek was swollen. (34 year-old mother)*

Similar stories were heard from local health workers:

*She was in the last trimester. She had gone to bring fodder from the forest. She slipped on the road, hurt her abdomen. She had bleeding and fainted. …Fortunately, the mother survived; however, the baby died. (Local Health Worker, HSP7)*

The heavy workload affected not only common women in the villages, but also the female health volunteers and auxiliary nurses due to work exhaustion:

*I slipped on the road when I was coming home with a jar of 10 litres on my back (with a strap across the head) and two jugs of five litres each in my hands. I fell on the ground. My belly bumped against the ground, and I started rolling down on the road. I had profuse bleeding after, and then delivered the babies. These were twins, both dead. (Auxiliary Nurse at a district meeting)*

#### 2.2 Daughter-in-law: A low voice, marginalised within a family

- ***➢ Mother-in-law and a daughter-in-law: ruler*** **versus** ***ruled***

The majority of women in this study were living in joint, two-generation families, a common tradition across Nepal. In the study villages, a daughter-in-law is always obliged to obey a mother-in-law’s decisions, including in matters such as her own and her baby’s health. Various accounts show that mothers-in-law perceive pregnancy and childbirth as everyday events requiring no special care. Therefore, it was beyond the expectation of daughters-in-law to get support from the family:

*Mothers-in-law tell that they also worked and gave birth to their boys and girls. They ask you, 'are you the only woman giving birth? We also gave birth'. Who cares that you need a rest, you need a good food? All that is expected from a daughter-in-law is work, work and work. (25 year-old mother)*

Many participants expressed the controlling nature of mothers-in-law. A local teacher described a daughter-in-law as being oppressed within a family to such an extent that her complaints are not heeded even when she is ill:

*Daughters-in-law are just the labourers within a family. Even when a daughter-in-law is pregnant, she has to cope with her mother-in-law’s regular complaints, and has to continue heavy work. When she tells about her illness, the mother-in-law often does not trust; mother-in-law thinks that she is telling a lie (Nauragareko). (Local teacher, SH4)*

During the fieldwork some families from the *Lama* community seemed to be a little more caring towards their daughters-in-law, at least immediately after birth. Yet, for a daughter-in-law, work is a customary norm, a key priority up to the last minute before birth.

- ***➢ Men’s involvement***

Husbands are not involved in matters related to their wives’ pregnancy and birth, as these are considered women’s business. Unless there is an emergency, men are not expected to engage in any of their wives’ work or provide them with any support during pregnancy and birth:

*My wife said, ‘You men can go out. Daughter-in-law is in labour pain (Kaitha). Even we women can't really do anything here. What can husbands (Lognes) do for a woman giving birth? (46 year-old man)*

Husbands are not expected to be present during childbirth. One mother, who was at home only with her husband when she had birthing pains throughout the day, did not tell him about this. Rather, she gave birth alone in the *Gotha* (cowshed). Unfortunately, she became unconscious after giving birth and could not cut her baby’s cord. The baby had already died:

*My husband was sleeping. There were no other people at home. I had pain from the morning and delivered the baby in the evening. After the pain became severe, I went inside Gotha. I became very weak and unconscious after giving birth. When I woke up, my baby was lying dead on the rice straw [only then the husband noted that she gave birth]. (18 year-old mother)*

Some husbands wish to support their wives but cannot negotiate it within their families. They are afraid of becoming a bad son in their mothers’ eyes. Another woman, who lost her first newborn last year, said her husband would wish to support her but feared whether it would insult his parents:

*I can't tell her to have a rest. It is up to my parents, my mother. I don’t like her to do a heavy work. However, I can’t really help. It is my mother who says who does what in our family. (19 year-old father)*

Talking to parents in favour of the wife is considered the sign of a weak man, a weak son who is teased as a henpecked husband. In a post interview chat, it was evident that this young father felt that he would be mocked by his neighbours and other men in the village if he started listening to his wife and supporting her in her day-to-day work.

A few women were neglected and verbally abused by their husbands and mothers-in-law after losing their babies, so they started living with their maternal family:

*It is very sad. Neither my husband nor my mother-in-law treats me well. He frequently shouted at me, and beat me. I don’t feel safe to go there. I am staying with my mother. I don’t know when I could return to my in-law’s home. (16 year-old mother)*

It was noted that these women accept the indifference and neglect from their husbands and mothers-in-law as their fate *(Bhagya).*

### 3. Perception of childbirth as a polluted event: Birth in *Gotha* (cowshed), and giving birth alone

The time around childbirth and after birth up to three weeks is perceived as a polluted period, locally called *Juthosutka*. During this time women are strictly forbidden to worship their God *(Dewata)*, and must avoid observing festivals and rituals. Such a perception of pollution and giving birth in *Gotha* is considered culturally safe in the villages at a time when policies discuss the need for skilled attendance and institutional births as strategies to address poor quality of care and high perinatal mortality [29, 30, 65, 66]. *Gotha* refers to the ground floor of a house where the family keeps cattle, goats, sheep and chicken. If the Gotha has any spare space, they also store firewood, grass and fodder. Women give birth in a corner of the *Gotha*, usually a short distance from the cattle. Some houses have a separate room on the ground floor, which they normally use for storing grass and firewood for the rainy season. It normally has only one entrance, little ventilation, and often no doors. The floor is usually damp and rough, and very cold in winter. Women give birth on old and worn-out dirty rugs, or they use dry straw on the floor.

In the study villages, a birthing woman is traditionally considered untouchable *(Chhuhi)* and left to give birth alone. Attendants, including family members, feel hesitant to touch a birthing woman and her baby. A mother who lost two babies soon after birth, described:

*Both times, I gave birth alone [in Gotha]. The first time, my sister-in-law witnessed, but she didn't touch me. She is a faith healer (Dhami); she worships Dewata (God). This time, my father’s sister (Fupu) and my mother were there but neither of them could touch me. I cut the baby’s cord, and buried the placenta myself. (19 year-old mother)*

Women are believed to be brave and courageous if they give birth alone and unaided. They are encouraged to hide their pain, remain quiet during labour, and not to let others notice it.

At the same time, women and their families feel it is physically and culturally safe to give birth in *Gotha* as it helps to keep the birthing woman and baby untouched from others and to prevent polluting a home and their *Dewata* (God). It was unfortunate that women did not even get birthing kits for hygiene as currently there is no more supply of these kits to this district, neither from government nor international aid agencies. It appeared that the health facilities stopped supplying them to more strongly discourage home/*Gotha* births.

With these beliefs about birth pollution in mind, it is perceived that God will be unhappy and will curse them if a woman gives birth inside the house, or enters the house before an auspicious day suggested by the local priest. During this confinement in *Gotha*, other family members have little contact with the mother and baby. Shivakumari’s baby died on the seventh day in the *Gotha*. Her mother-in-law was hesitant to enter *Gotha* to assist Shivakumari:

*We have God (Dewata) inside our house. It is not good to see and touch a birthing woman. When my daughter-in-law’s labour started, I was inside this house, over there (she points to a place near the kitchen). Daughter-in-law went inside the Gotha. My younger daughter (14 years old) went frequently to see her inside the Gotha.*

*...I don't know how that baby died. I didn't go to Gotha. I was waiting to see the baby once my daughter-in-law entered the home. (Shivakumari’s mother-in-law)*

Observing the pollution belief *(Chhuhi)* during menstruation and birth is considered a religious ritual *(Dharma)* by local faith healers. Faith healers remain careful enough not to physically touch the woman and her baby to avoid being polluted:

*When a woman is giving birth, I sprinkle rice grains and do Mantra (Paturne) from a distance. I immediately leave the birthing place after the baby comes out, that is pollution (Chhuhi). I can’t touch the mother and baby. I can touch them only after the pollution [period] is over.*

Such a perception about pollution has affected birthing women to such an extent that they did not receive support even in high risk situations. A local auxiliary nurse described her experience with a woman giving birth on the way to a birthing unit, who was bleeding while others just watched on:

*I saw a woman who delivered her baby on her way to the birthing centre. She was bleeding. There were other women, but they were all just watching her from a little distance. I used polythene bags as gloves. I removed her placenta. The birthing centre was still an hour away. We then made a stretcher out of a shawl (Sal) and requested them to carry her to the birthing centre. (Senior Auxiliary Nurse, HCM3)*

Providing childbirth services was not favoured by some health volunteers and support staff because of a hesitancy to touch birthing women due to their religious beliefs.

#### 3.1 Child gender as a factor to negotiate birth pollution

The length of the pollution period varies between a newborn girl and boy. In the study communities a woman who bears a boy is considered less polluted and can enter the home as early as the sixth day to perform *Chhaith* – a ritual performed usually on the sixth day after birth. It is a common belief among the Hindus that the Goddess Saraswati (Goddess of wisdom) will visit the baby on this auspicious day and will create a destiny (a life script) for the baby’s future. The ritual is observed by lighting an earthen lamp, putting a pen and paper at the baby’s side and singing (traditional songs – *Bhajan*) and dancing throughout the evening and the night of the sixth day. However, the pollution period for a newborn girl *(Chhoriko chhuhhi)* is longer. In the study villages, *Chhaith* is celebrated only for a newborn boy and was considered to bring good luck to the baby. This is not performed for girls because families do not consider it worth spending money on observing *Chhaith* for girls. The naming ceremony is also performed only for the boys, varying between the ninth and eleventh day after birth; girls are simply named informally by the parents because a formal ceremony is not considered worthwhile for a girl.

In addition to gendered constructions as discussed above, it was observed during the fieldwork that there exists a potential for negotiating the space for birthing outside the house (for example, in the community birthing centre) if the house is very small with no spare rooms for birthing and if even the *Gotha* is too small. Confinement in the *Gotha* was also influenced by the easier access of basic amenities such as water supply and toilets, which are located on the ground floor and women do not have to climb difficult (and unsafe) ladders if the basic amenities were located inside the house on the first floor. Such amenities are not necessarily available in the formal birthing centres.

## Discussion

Gendered constructions of motherhood emerged as major factors which contribute to perpetuating the pattern of high perinatal death rates in these mountain villages. Women’s experiences and perceptions revealed that pregnancy, childbirth and postnatal care are complicated phenomena in the complex interrelated gendered constructions of *Gharbar* (girl settlement)*,* the weak social position of a daughter-in-law, and the perception of childbirth and the postnatal period as a time of ritual pollution.

### *Gharbar* (girl settlement): Gendered motive underlying child marriages and son preference

**Fig. 1:**
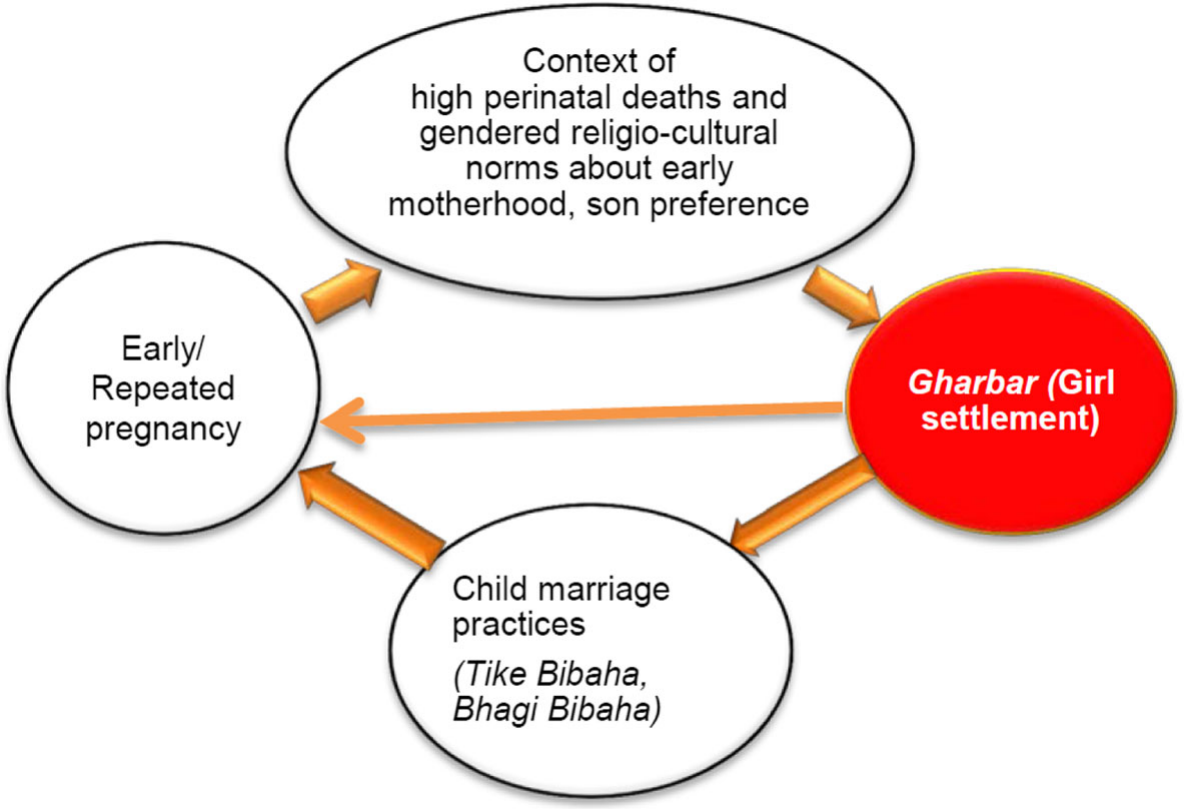
Vicious loop of repeated pregnancy and baby loss in the study villages

The first theme discussed gendered social constructs, namely child marriage, repeated childbearing and son preference, which make women and babies vulnerable to poor perinatal survival. Child marriages and son preference are common in many developing countries and a range of studies describe a high prevalence of child marriages [67, 68] and son preference [69–73] in South Asia as socio-cultural factors contributing to poor maternal and child survival. In discussing son preference, other studies have described sex-selective abortions, neglect of women and girls, and discrimination against females by withholding care from newborn girls due to son preference, as commonly practised in South Asian countries [70, 71, 73]. In the villages in this study, similar beliefs and practices exist but in addition the construct of *Gharbar* ‘settling a daughter down’ is a major driving factor which perpetuates a vicious cycle of early marriage, and early and repeated childbearing (often closely spaced) with the strong desire for a surviving baby boy (Fig. 1).

The tradition of *Gharbar* described in this study is consistent with practices reported in India in studies describing the socio-cultural context of marriage survival and parenthood [74–76]. Having a child (motherhood) is compulsory in Indian societies, which is seen as a factor enhancing a woman’s status, and cementing the bonds of marriage between spouses. Patel (2006) described how a childless woman feels anxious, has low self-esteem, becomes powerless and in most cases faces neglect and divorce. In this study, the drive towards adolescent girls’ settlement is also linked to similar pressures for early motherhood and bearing babies at a young age, and further pressures for immediate child bearing when a woman loses a baby and has no baby boy yet. Rather than just counting the babies’ deaths, it is more about repeated childbearing in the hope of having a surviving baby, and thereby settling in a *Gharbar* to secure the marriage and feel secure in the in-laws’ family.

Likewise, the context of girl settlement in this study resembles the description by Ware [77] of the life cycle of a typical woman in many traditional societies. Ware described the importance of the adolescent stage of a girl in developing countries with respect to protecting virginity, entering the boy’s family (through early marriage) to contribute to agriculture and household duties, and to bear children, preferably boys (sons) early so that she can feel secure in her present life and in older age. More than 35 years later, Ware’s findings still hold true in the remote mountain villages of Nepal, and although Ware’s description pertains to the quality of women’s reproductive life in general, the present study from Nepal adds that it is crucial to bring about changes in the components of *Gharbar* if we are to address the continuing high levels of perinatal mortality.

### Pregnancy and childbirth in the intra-familial dynamics of relationships and power

**Fig. 2:**
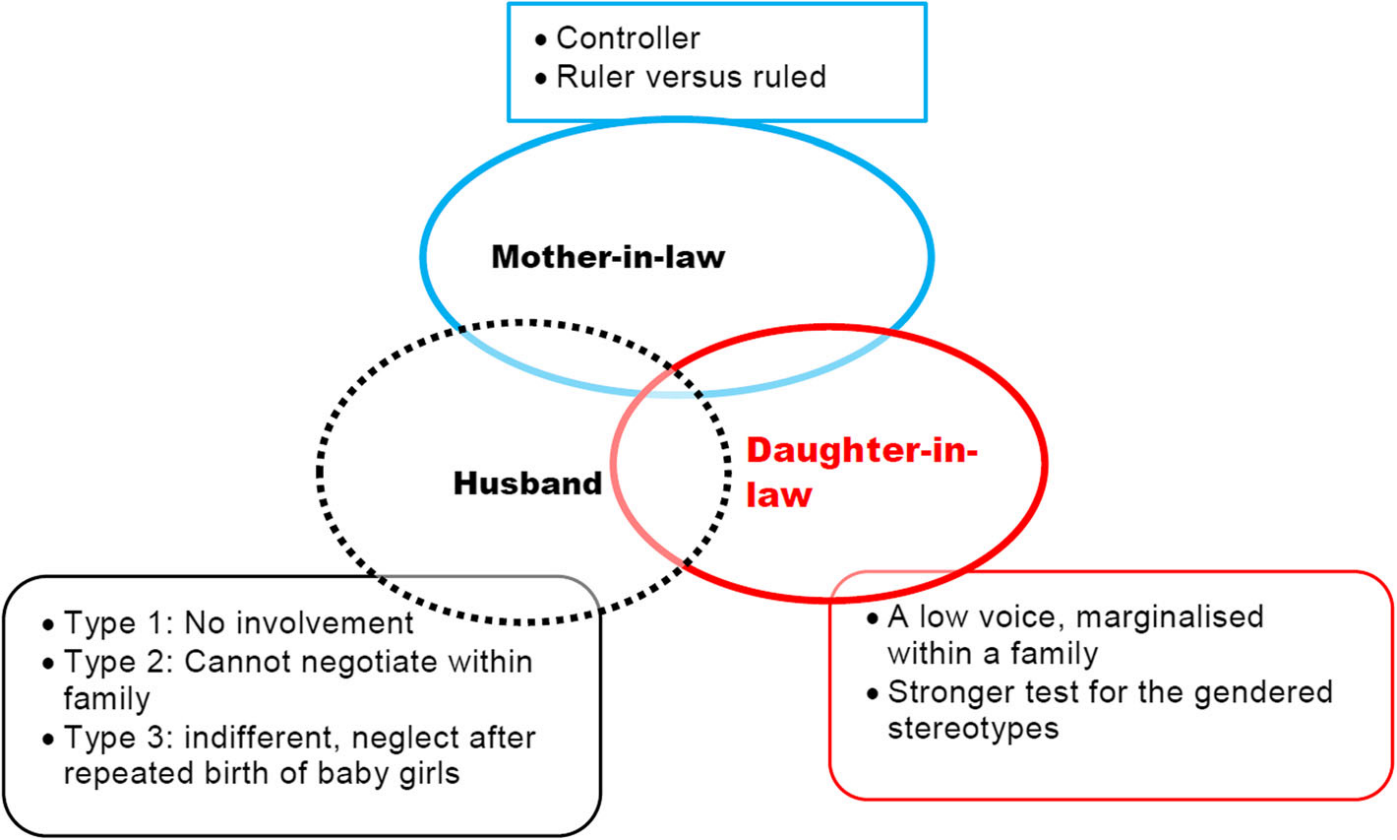
Daughter-in-law in family dynamics of relationships and power

The study identified a gendered intra-familial context as a crucial factor shaping access to healthcare during pregnancy, childbirth and the postnatal period for a daughter-in-law and her baby. The villages follow the extended family system with typically two generations comprising the father, mother and their married sons. In such families, the position of a daughter-in-law is part of a hierarchy-based complex triad of intra-familial relationship dynamics: the daughter-in-law herself, her husband and her mother-in-law (Fig. 2).

In this study, the intra-familial gendered role construction is particularly responsible for not seeing pregnancy and childbirth as special events worthy of providing additional care. Firstly, these are viewed as a woman’s duty, which should require no special exemption from work– even heavy physical work, and secondly, a daughter in-law is at a double disadvantage (a woman suffering from another woman) due to the negative controlling role of the mother-in-law, often preventing access to even basic healthcare. All of these make young girls and their babies vulnerable to sickness and death. Birth preparedness, although discussed in policies [30], is practically non-existent in such a context. Besides finding ways to change the attitudes of mothers-in-law to be more supportive of their daughters, as has been achieved in some Indigenous cultures elsewhere [78], finding socially acceptable ways for the community to encourage men’s interest and involvement in pregnancy and childbirth should be given a strong priority for improving maternal and perinatal health; both of these have been achieved for Indigenous cultures elsewhere [78, 79].

In many South Asian and Sub-Saharan African countries, mothers-in-law are the power bearers in families to decide access to healthcare [80], and this was no different in the present study. Previous studies from Nepal [24, 41, 46] described the crucial role of mothers-in-law in their daughters-in-laws’ pregnancy and childbirth. Kaphle [41] described mostly positive roles of mothers-in-law in care and support during pregnancy and childbirth. It appears that Kaphle et al. did not identify tension between daughters-in-law and mothers-in-law regarding accessing healthcare, as was found this in this study. Similar to Simkhada et al. (2010) and Shrestha et al. (2012), the present study noted that mothers-in-law are often seen in negative controller roles, often imposing their own experiences of pregnancy and childbirth on their daughters-in-law, however difficult those experiences might have been. It may be that in these communities, becoming a mother-in-law is the only chance a woman has to show her pride, status and power, and she does this also by reinforcing the status quo of how she has experienced her own pregnancies, births, and losses. At the same time, the prominent role of mothers-in-law in maintaining this hierarchy is perhaps not surprising among women who themselves were never treated equal to men or anyone in their family until they became a mother-in-law.

None of the previous studies from Nepal [24, 41, 46] specifically has discussed men’s role in pregnancy and childbirth. The present study confirms that husbands were rarely involved in matters related to pregnancy and childbirth, even though national and international policies such as ICPD recommend male participation in reproductive health [37]. It is known that in many developed countries, men’s engagement in pregnancy and childbirth has been enhanced through parents’ group classes, and encouragement to stay together and support their partners during labour [81]. As evident in WHO (2007), 95% of prospective fathers in England and Denmark in the mid-1990s were by the side of their wives during labour at the hospital. Developing countries, particularly their low socioeconomic areas are far behind developed countries in terms of male participation in supporting their pregnant and postnatal wives. In the study villages pregnancy and birth are strongly maintained as only women’s business, and men who wanted to help were dissuaded from doing so for fears of social stigmatisation. Men in the study villages are not expected to stay at home during a woman’s childbirth; neither are they allowed to stay with their wives if they deliver at a health facility, thus clearly distancing them from childbirth related matters. Men who listen to their wives are not considered ‘good sons’ in their mother’s eyes, and those providing care and support to their wives are often criticised in the communities as weak, or a “womanly man” *(Joitingre).* Therefore, such gendered status of men proves to be a barrier to engage them in pregnancy and childbirth related matters.

On the other hand, in Asia and Africa, men’s involvement in maternal and child health should be considered crucial for reducing maternal and infant mortality by adopting family planning practices and increasing women’s access to healthcare during obstetric emergencies [81, 82]. However, in the present study, family planning would be seen to work counter to the cultural desire for a woman to bear a surviving child, preferably a son as quickly as possible. Besides a general reluctance to use contraceptives due to high infant mortality and desire for sons, it is speculated that the stronger aversion to vasectomy is also related to male identity *(Purushatwa)* which is mainly about proving a man’s ability to father children. Men are considered to lose their male identity and become weak after having had a vasectomy.

### Childbirth as a polluted event: Birth in *Gotha* (cowshed), and giving birth alone

**Fig. 3:**
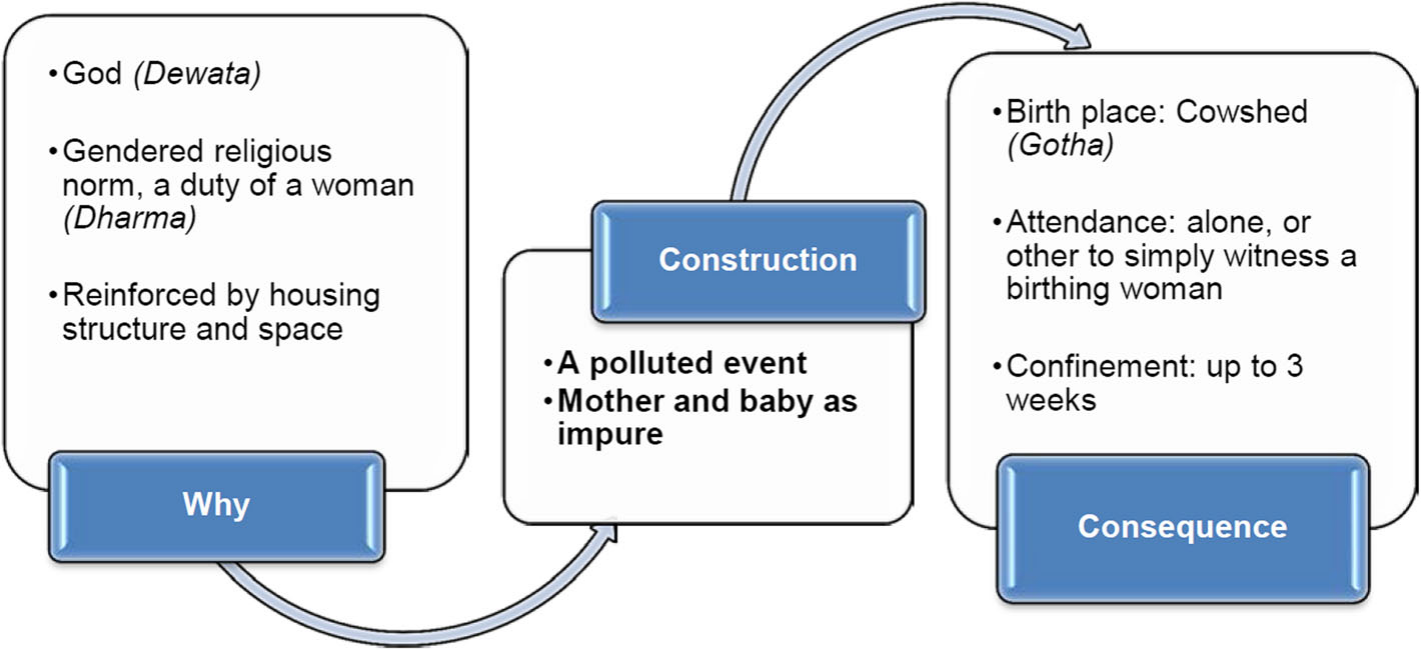
Perception of childbirth as a polluted event

Segregation or confinement during the postnatal period and considering childbirth as a form of pollution is noted in previous studies from India [83], Bangladesh [84–86], and in Nepal [41, 87]. Bandyopadhyay described that the confinement of mother and baby after childbirth is a cultural tradition in India, followed due to a belief that the placental/postnatal blood is impure. The birthing woman herself is believed to be in danger—in an inauspicious period, and can infect/pollute others. Another reason for confinement is believed to be to isolate the baby from visitors to protect from infection. Confinement during menstruation and childbirth/postnatal period is considered as a form of temporary pollution, whereas the belief that lower caste people are not pure, particularly for holy rituals and should not touch a person belonging to an upper caste is described as a permanent form of pollution. Studies from Bangladesh describe the locals keeping both mother and baby strictly confined, generally for up to 9 days (which can sometimes go up to 40 days) in the place of birth is to protect the mother and baby from malicious spirits. Previous studies in Nepal describe childbirth pollution as ritual tradition followed by the locals that makes them feel culturally safe during and after birth [41], and a period during which the locals consider that it is essential to confine women to heal themselves, as they are perceived to have become weak after childbirth. These studies have ignored to look into the fact that such practices at the same time deny a woman and her baby access to even basic healthcare. The present study highlights the fact that due to the perception of birth as a polluted event, the occurrence of childbirth in the *Gotha* (cowshed) in the study villages is nowhere near the common international debate about ‘home versus health facility births’ or ‘unskilled (lay attendants) versus skilled attendants’. The present study reveals that the practice of ritual pollution not only confines a woman and her baby in the *Gotha*, but also compels women to give birth unaided and isolated without letting others touch the delivering mother (Fig. 3), both to protect others from pollution but also as a sign of strength for the woman to give birth alone. Perinatal deaths during this period of isolation are matters of low concern, and often rationalised as *Lekhanta* (destiny) [61, 88] The context created by the above beliefs and practices in these villages is therefore likely to be a social barrier to seeking healthcare and help maintain the high vulnerability of newborn babies to poor perinatal survival.

Medical evidence shows that the time around birth is the most critical time to save the lives of a large number of mothers and babies [1, 89]. Nepal’s health policies and other studies [12, 45, 46] encourage women to deliver in health facilities to avoid potential risks of infection and death, especially during obstetric emergencies. A skilled attendant at childbirth is considered essential even if it is a homebirth. Far from delivering in a health facility (birthing centre) with skilled birth attendants which the national health policies aim for [30, 65], the present study found that women end up delivering, not even in a clean place at their home, but they deliver alone and unaided in *Gotha* (cowshed), which for most purposes is hardly clean, safe and comfortable. Seeking healthcare at health facilities and being attended at delivery by skilled birth attendants are completely compromised during this “polluting” period.

### Implications of the study

Findings of the present study show that recommending quick-fix medical solutions alone will not be sufficient to reduce stillbirth and neonatal mortality rates in remote Nepal. The context of poor perinatal survival is rather complex, and therefore, it is not enough just to introduce policies to get rid of adverse behaviours which have a very strong social and cultural basis. Policy makers and programme implementers must acknowledge and understand the power of this intra-familial context which has shaped women’s pregnancy and childbirth experiences in the villages for ages. Understanding the intra-familial context is vital to leveraging the effectiveness of family and community based interventions. Engagement with family members, particularly mothers-in-law and husbands is an important part of community engagement strategies. Community based maternal and child health programmes should not just target pregnant women (the daughters-in-law). In addition, they should strongly engage mothers-in-law because disengaging mothers-in-law could be perceived as a challenge by them who fear the loss of their power, and which may ultimately make them further controlling and hostile towards their daughters-in-law. Likewise, keeping men disengaged from their wives’ pregnancy and childbirth would perpetuate a gender stereotype that pregnancy and childbirth are women’s only matters. Ignoring the possibility of behaviour change among the mothers-in-law and husbands to play positive roles in pregnancy and childbirth of wives/daughters-in-law, any family and the community based intervention on maternal and newborn health, as suggested in a range of studies [13, 16, 90] is much less likely to succeed.

Ensuring men’s involvement with their wives during labour, both at home birth and health facility births could be part of future interventions. Likewise, rather than focussing on current women’s only groups, namely the ‘Mothers Group’ [91], mixed sex groups could be beneficial to reduce gender stereotypes and to sensitise men in supporting pregnancy and childbirth. Such mixed-group approaches have not been tried before in the study area, or in Nepal as a whole. Therefore as a first step, it is recommended that policies and programmes are revisited such as mobilisation of only female health volunteers in Nepal [91], and group based education to only pregnant women’s groups. It appears that mobilising ‘women’s only groups’ in the long run have reinforced gendered stereotypes of pregnancy, childbirth and newborn care as women’s only responsibilities.

Maternal and newborn health policies and programmes should not only target in prescribing tablets—the basic medicines [61] and treating mother and child complications once they are already sick. The complexity of socio-cultural contexts where these women live, work and experience their motherhood demands solutions beyond simple medical approach. The health systems, more specifically primary health care approach should be strongly oriented towards a social determinant of health approach [92] so that actions and approaches which emerge at grass roots could be helpful to change health promotive behaviours throughout pregnancy, childbirth and postnatal period. Local primary health care programmes should be strongly driven to prevent social determinants such as child marriages and son preference. Reducing poverty, empowering women, and access to quality education for girls are frequently referred as the most effective interventions to prevent child marriages and son preference [77, 93]. Besides a long-term focus on women empowerment and education for girls, participatory interventions mobilising women’s group [94, 95], and local stakeholders’ group [96], which are proven intervention to tackle high perinatal mortality could be utilised to educate communities to prevent discriminatory norms and values about child marriage and son preference, and thereby break the motive of *Gharbar* to prevent the vicious loop of ongoing perinatal deaths in the mountain villages. Inter-sectoral efforts at different settings (schools, health facilities, parent groups, faith/religious groups) may be useful to break the notion of the *Gharbar* (girl settlement) that perpetuates the loop of child marriages, early and repeated pregnancies, and persistent perinatal deaths in the villages. It is this construct of girl settlement backed up by the discriminatory norms and values that makes it so powerful that child marriages and son preferences continue in the villages despite legal measures [93, 97]. It has contributed to the persistence of vulnerabilities to perinatal deaths, has let this vulnerability remain unchecked, such as in the villages by not registering any marriages, births and deaths before they reach legal age. Making every death count and visible is one of the strategic actions strongly emphasised in maternal and neonatal health policies [10, 98]. However, policy or legislative changes alone appeared less impactful unless changes are brought from within the communities themselves.

Given the near impossibility of changing people’s mindset about birth pollution and delivering the baby away from the main living quarters of the house, funds should be directed towards creating more birthing centres and ensuring the deployment of trained female health workers in safe working conditions so that pregnant women are able to deliver in clean and safe conditions, attended by trained birth attendants. At the same time, intense and continuous behaviour change communications should be conducted to change the current mindset of mothers-in-law and the husbands and make them value each pregnancy and each child birth as very precious. Sympathetic religious leaders who outrank local faith healers should be called upon to visit these villages from time to time to talk to the villagers (especially the mothers-in-law) to change their current mindset. Above all, education (through informal means if necessary) should be imparted to all, so that people can understand that they can change their *Bhagya (Fate)* and *Lekhanta (Destiny)*.

### Limitation of the study

The qualitative methodology of this study confines us to elucidate gender related cultural contexts in the isolated mountain villages of Nepal. The study design no longer helps to establish any causal relationships. However, the social constructions of gender which emerged in this study could be inferred to other parts of Nepal and other developing countries grappling with high perinatal deaths.

## Conclusions

Discussing gender with mere disaggregation of sickness and death as a dichotomy between baby boys and girls will have less impact in addressing poor perinatal survival in the rural villages. This study argues that poor perinatal survival in developing countries such as Nepal cannot be understood without understanding gendered constructions along the pre-pregnancy, postnatal continuum from being a girl *(Gharbar),* a pregnant mother (daughter-in-law), and a woman and baby at and after birth (ritual pollution). Perinatal survival should be seen as a ‘social construction of gender’, thus not just limiting it to an indicator of quality of medical care, which predominantly values a medical construction and hence medical intervention. Only then, can we see the complex gendered contexts reflected in service planning and programme implementation. This will also facilitate the formulation and implementation of appropriate policies, thus moving away from merely acknowledging gender equality on paper, and from the study of mere distribution of sickness and death by sex in national reports.

## Additional files

Additional file 1: Study Participants (DOCX 19 kb)

Additional file 2: In-depth Interview Guides (DOCX 27 kb)

## Abbreviations

CSDH: Commission on Social Determinants of Health
LSTM: Liverpool School of Tropical Medicine
MOHP: Ministry of Health and Population
NDHS: Nepal Demographic and Health Survey.
NHMRC: National Health and Medical Research Council

## References

[1] Lawn JE, Blencowe H, Oza S, You D, Lee ACC, Waiswa P (2014). Every newborn: progress, priorities, and potential beyond survival. Lancet.

[2] WHO (2000). Definitions and indicators in family planning maternal & child health and reproductive health used in the WHO regional office for Europe.

[3] MacDorman M, Gregory E (2015). Fetal and perinatal mortality: United States, 2013. Natl Vital Stat Rep.

[4] Monk A, Harris K, Donnolley N, Hilder L, Humphrey M, Gordon A (2016). Perinatal deaths in Australia, 1993–2012. Perinatal deaths series no. 1. Cat. No. PER 86.

[5] Blencowe H, Cousens S, Jassir FB, Say L, Chou D, Mathers C (2016). National, regional, and worldwide estimates of stillbirth rates in 2015, with trends from 2000: a systematic analysis. Lancet Global Health.

[6] UNICEF (2015). Levels and trends in child mortality: report 2015, estimates developed by the UN inter-agency Group for Child Mortality Estimation.

[7] WHO (2017). Maternal, newborn, child and adolescent health: stillbirths.

[8] WHO (2017). Global Health Observatory (GHO) data, neonatal mortality: situation and trends: World Health Organization.

[9] Lawn JE, Blencowe H, Waiswa P, Amouzou A, Mathers C, Hogan D, et al. Stillbirths: rates, risk factors, and acceleration towards 2030. Lancet. 2016;387(10018):587–603. 10.1016/S0140-6736(15)00837-5 26794078

[10] UNICEF and WHO. Countdown to 2015 maternal, newborn and child survival: a decade of tracking progress for maternal, newborn and child survival, the 2015 report. Geneva: World Health Organization; 2015.

[11] MOHP, New ERA, ICF International Inc (2012). Nepal demographic and health survey 2011.

[12] Lawn JE, Cousens S, Zupan J (2005). 4 million neonatal deaths: when? Where? Why?. Lancet.

[13] Darmstadt GL, Bhutta ZA, Cousens S, Adam T, Walker N, de Bernis L (2005). Evidence-based, cost-effective interventions: how many newborn babies can we save?. Lancet.

[14] Bhutta ZA, Yakoob MY, Lawn JE, Rizvi A, Friberg IK, Weissman E (2011). Stillbirths: what difference can we make and at what cost?. Lancet.

[15] Lawn JE, Blencowe H, Pattinson R, Cousens S, Kumar R, Ibiebele I (2011). Stillbirths: where? When? Why? How to make the data count?. Lancet.

[16] Bhutta ZA, Das JK, Bahl R, Lawn JE, Salam RA, Paul VK (2014). Can available interventions end preventable deaths in mothers, newborn babies, and stillbirths, and at what cost?. Lancet.

[17] Thaddeus S, Maine D (1994). Too far to walk: maternal mortality in context. Soc Sci Med.

[18] Mosley WH, Chen LC (1984). An analytical framework for the study of child survival in developing countries. Popul Dev Rev.

[19] CSDH (2008). Closing the gap in a generation: health equity through action on the social determinants of health. Final report of the commission on social determinants of health.

[20] LSTM (1999). Guidelines for the analysis of gender and health.

[21] Pokhrel S, Snow R, Dong H, Hidayat B, Flessa S, Sauerborn R (2005). Gender role and child health care utilization in Nepal. Health Policy.

[22] Mahy M (2003). Childhood mortality in the developing world: a review of evidence from the demographic and health surveys.

[23] Brunson J (2010). Confronting maternal mortality, controlling birth in Nepal: the gendered politics of receiving biomedical care at birth. Soc Sci Med.

[24] Simkhada B, Porter MA, van Teijlingen ER (2010). The role of mothers-in-law in antenatal care decision-making in Nepal: a qualitative study. BMC Pregnancy Childbirth..

[25] Silverman JG, Decker MR, Cheng DM, Wirth K, Saggurti N, McCauley HL (2011). Gender-based disparities in infant and child mortality based on maternal exposure to spousal violence: the heavy burden borne by Indian girls. Archives Pedia Adolescent Med..

[26] Martines J, Paul VK, Bhutta ZA, Koblinsky M, Soucat A, Walker N (2005). Neonatal survival: a call for action. Lancet.

[27] de Bernis L, Kinney MV, Stones W, ten Hoope-Bender P, Vivio D, Leisher SH (2016). Stillbirths: ending preventable deaths by 2030. Lancet.

[28] Mason E, McDougall L, Lawn JE, Gupta A, Claeson M, Pillay Y (2014). From evidence to action to deliver a healthy start for the next generation. Lancet.

[29] MOHP (2004). National Neonatal Health Strategy. Child health division DoHS, Nepal.

[30] MOHP (2006). National Safe Motherhood and newborn health long-term plan, 2006–2017. Family health division DoHS, Nepal.

[31] Ulizzi L, Zonta L (2002). Sex differential patterns in perinatal deaths in Italy. Hum Biol.

[32] Fuse K, Crenshaw EM (2006). Gender imbalance in infant mortality: a cross-national study of social structure and female infanticide. Soc Sci Med.

[33] Arnold F. Sex preference and its demographic and health implications. Int Fam Plan Perspect. 1992;18:93–101.

[34] Nie J-B (2011). Non-medical sex-selective abortion in China: ethical and public policy issues in the context of 40 million missing females. Br Med Bull.

[35] Shah R, Mullany LC, Darmstadt GL, Talukder RR, Rahman SM, Mannan I (2014). Determinants and pattern of care seeking for preterm newborns in a rural Bangladeshi cohort. BMC Health Serv Res.

[36] Rosenstock S, Katz J, Mullany LC, Khatry SK, LeClerq SC, Darmstadt GL (2013). Sex differences in neonatal mortality in Sarlahi, Nepal: the role of biology and environment. J Epidemiol Community Health.

[37] UNFPA (2014). Programme of action of the international conference on population development, 20th anniversary edition.

[38] Stenberg K, Axelson H, Sheehan P, Anderson I, Gülmezoglu AM, Temmerman M (2014). Advancing social and economic development by investing in women's and children's health: a new global investment framework. Lancet.

[39] UNFPA (2013). The state of the world population 2013: motherhood in childhood: facing the challenge of adolescent pregnancy.

[40] Bloom SS, Wypij D, Gupta MD (2001). Dimensions of women’s autonomy and the influence on maternal health care utilization in a north Indian city. Demography.

[41] Kaphle S, Hancock H, Newman LA (2013). Childbirth traditions and cultural perceptions of safety in Nepal: critical spaces to ensure the survival of mothers and newborns in remote mountain villages. Midwifery.

[42] Dhakal S, van Teijlingen E, Raja EA, Dhakal KB (2011). Skilled Care at Birth among rural women in Nepal: practice and challenges. J Health Popul Nutr.

[43] Deo KK, Paudel YR, Khatri RB, Bhaskar RK, Paudel R, Mehata S (2015). Barriers to utilization of antenatal Care Services in Eastern Nepal. Front Public Health.

[44] Paudel M, Khanal V, Acharya B, Adhikari M. Determinants of postnatal service utilization in a Western District of Nepal: community based cross sectional study. J Women’s Health Care. 2013;2(126):2167–0420.

[45] Wagle RR, Sabroe S, Nielsen BB (2004). Socioeconomic and physical distance to the maternity hospital as predictors for place of delivery: an observation study from Nepal. BMC Pregnancy Childbirth..

[46] Shrestha SK, Banu B, Khanom K, Ali L, Thapa N, Stray-Pedersen B (2012). Changing trends on the place of delivery: why do Nepali women give birth at home?. Reprod Health.

[47] Shah R, Rehfuess EA, Maskey MK, Fischer R, Bhandari PB, Delius M (2015). Factors affecting institutional delivery in rural Chitwan district of Nepal: a community-based cross-sectional study. BMC Pregnancy Childbirth.

[48] Dahal RK (2013). Factors influencing the choice of place of delivery among women in eastern rural Nepal. Int J Maternal Child Health.

[49] Manandhar S, Ojha A, Manandhar D, Shrestha B, Shrestha D, Saville N (2010). Causes of stillbirths and neonatal deaths in Dhanusha district, Nepal: a verbal autopsy study. Kathmandu Univ Med J (KUMJ)..

[50] Manandhar S, Manandhar D, Adhikari D, Shrestha J, Rai C, Rana H (2015). Analysis of health facility based perinatal verbal autopsy of electoral constituency 2 of Arghakhanchi District, Nepal. J Nepal Health Res Counc.

[51] Shrestha M, Manandhar DS, Dhakal S, Nepal N (2006). Two year audit of perinatal mortality at Kathmandu medical college teaching hospital. Kathmandu Univ Med J (KUMJ).

[52] Khanal S, Sharma J, Gc VS, Dawson P, Houston R, Khadka N (2011). Community health workers can identify and manage possible infections in neonates and young infants: MINI--a model from Nepal. J Health Popul Nut.

[53] Dhakwa JR, Bhandari NN, Shedain PR, Khanal S, Pradhan A, Shrestha BM (2014). A report on verbal autopsy to ascertain causes of neonatal deaths in Nepal 2014.

[54] Polit DF, Beck CT. Nursing research: generating and assessing evidence for nursing practice. 9th ed. Philadelphia: Wolters Kluwer Health, Lippincott Williams & Wilkins; 2012.

[55] Liamputtong P (2007). Researching the vulnerable: a guide to sensitive research methods. London.

[56] NPC, UNDP (2014). Nepal human development report 2014: beyond geography, unlocking human potential.

[57] Ritchie J, Lewis J, Nicholls CM, Ormston R (2003). Qualitative research practice: a guide for social science students and researchers: sage.

[58] Patton MQ (2002). Qualitative interviewing. Qualitative research and evaluation methods. 3rd ed.

[59] Devers KJ, Frankel RM (2000). Study design in qualitative research--2: sampling and data collection strategies. Educ Health.

[60] Braun V, Clarke V (2006). Using thematic analysis in psychology. Qual Res Psychol.

[61] Paudel M (2017). Socio-cultural and health care contexts of perinatal survival in Rural Mountain villages of Nepal: Flinders University.

[62] NHMRC (2015). National statement on ethical conduct in human research 2007 (updated may 2015).

[63] MOHP (2011). Nepal population report 2011. Division P.

[64] Karki S. A study on dowry related violence in Nepal. Kathmandu; 2014.

[65] MOHP (2006). National Policy on skilled birth attendants (Supplementatry to safe motherhood policy 1998). In: family health division DoHS, Nepal, editor.

[66] MOHP (2013). Mother’s protection program-implementation guideline, 2008 (second amendment 2013). In: Department of Health Services N, editor.

[67] Maharjan RK, Karki KB, Shakya TM, Aryal B (2012). Child marriage in Nepal: a research report.

[68] Sharma V, Katz J, Mullany LC, Khatry SK, LeClerq SC, Shrestha SR (2008). Young maternal age and the risk of neonatal mortality in rural Nepal. Arch Pedia Adol Med.

[69] Almond D, Edlund L, Milligan K (2013). Son preference and the persistence of culture: evidence from south and east Asian immigrants to Canada. Popul Dev Rev.

[70] Arnold F, Choe MK, Roy TK (1998). Son preference, the family-building process and child mortality in India. Popul Stud.

[71] Barot S (2012). A problem-and-solution mismatch: son preference and sex-selective abortion bans. Gutmacher Policy Rev.

[72] Brunson J (2010). Son preference in the context of fertility decline: limits to new constructions of gender and kinship in Nepal. Stud Fam Plan.

[73] Pande R, Malhotra A, Mathur S, Mehta M, Malhotra A, Lycette MA (2006). Son preference and daughter neglect in India: what happens to living girls?.

[74] Riessman CK (1995). Locating the outsider within: studying childless women in India. Reflections.

[75] Patel T (2006). Fertility behaviour: population and society in a Rajasthan village.

[76] Widge A (2002). Sociocultural attitudes towards infertility and assisted reproduction in India.

[77] Ware H (1981). Women, demography and development (demography teaching notes; 3).

[78] Lowell A, Kildea S, Liddle M, Cox B, Paterson B (2015). Supporting aboriginal knowledge and practice in health care: lessons from a qualitative evaluation of the strong women, strong babies, strong culture program. BMC preg childbirth.

[79] Government of Nunavut (2009). Nunavut maternal and newborn health care strategy 2009–2014.

[80] Hussein J, McCaw-Binns A, Weber R (2012). Maternal and perinatal health in developing countries.

[81] WHO (2007). Fatherhood and health outcomes in Europe.

[82] Piet-Pelon NJ, Rob U, Khan M (1999). Men in Bangladesh, India, and Pakistan: reproductive health issues: Hindustan publishing corporation (India).

[83] Bandyopadhyay M (2009). Impact of ritual pollution on lactation and breastfeeding practices in rural West Bengal. India Int Breastfeeding J.

[84] Winch PJ, Alam MA, Akther A, Afroz D, Ali NA, Ellis AA (2005). Local understandings of vulnerability and protection during the neonatal period in Sylhet District, Bangladesh: a qualitative study. Lancet.

[85] Tarafder T, Sultan P (2014). Reproductive health beliefs and their consequences: a case study on rural indigenous women in Bangladesh. Australasian J Reg Stud.

[86] Darmstadt GL, Syed U, Patel Z, Kabir N (2006). Review of domiciliary newborn-care practices in Bangladesh. J Health Popul Nutr.

[87] Sharma S, van Teijlingen E, Hundley V, Angell C, Simkhada P (2016). Dirty and 40 days in the wilderness: eliciting childbirth and postnatal cultural practices and beliefs in Nepal. BMC Preg Childbirth.

[88] Paudel M, Javanparast S, Dasvarma G, Newman L (2018). Religio-cultural factors contributing to perinatal mortality and morbidity in mountain villages of Nepal: implications for future healthcare provision. PLoS One.

[89] Dickson KE, Kinney MV, Moxon SG, Ashton J, Zaka N, Simen-Kapeu A (2015). Scaling up quality care for mothers and newborns around the time of birth: an overview of methods and analyses of intervention-specific bottlenecks and solutions. BMC Preg Childbirth..

[90] Bhutta ZA, Darmstadt GL, Hasan BS, Haws RA (2005). Community-based interventions for improving perinatal and neonatal health outcomes in developing countries: a review of the evidence. Pediatrics.

[91] MOHP (2010). Community based newborn care program (CB-NCP): program management module (first Ammendment). Child health division DoHS, Nepal.

[92] Irwin A, Scali E, Irwin A, Scali E (2010). Action on the social determinants of health: learning from previous experiences. Social determinants of health discussion paper 1 (debates).

[93] Human Rights Watch (2016). Our time to sing and play child marriage in Nepal.

[94] Manandhar DS, Osrin D, Shrestha BP, Mesko N, Morrison J, Tumbahangphe KM (2004). Effect of a participatory intervention with women's groups on birth outcomes in Nepal: cluster-randomised controlled trial. Lancet.

[95] O'Rourke K, Howard-Grabman L, Seoane G (1998). Impact of community organization of women on perinatal outcomes in rural Bolivia. Revista Panamericana De Salud Pública =. Pan Am J Public Health.

[96] Persson LA, Nga NT, Malqvist M, Thi Phuong Hoa D, Eriksson L, Wallin L (2013). Effect of facilitation of local maternal-and-newborn stakeholder groups on neonatal mortality: cluster-randomized controlled trial. PLoS Med.

[97] DoHS (2016). Safe abortion service program implementation guideline 2016. Family health division DoHS, Nepal.

[98] WHO (2014). Every newborn: an action plan to end preventable deaths.

